# Technical development of PubMed Interact: an improved interface for MEDLINE/PubMed searches

**DOI:** 10.1186/1472-6947-6-36

**Published:** 2006-11-03

**Authors:** Michael Muin, Paul Fontelo

**Affiliations:** 1 Office of High Performance Computing and Communications, National Library of Medicine, 8600 Rockville Pike, Bethesda, Maryland 20894, USA

## Abstract

**Background:**

The project aims to create an alternative search interface for MEDLINE/PubMed that may provide assistance to the novice user and added convenience to the advanced user. An earlier version of the project was the 'Slider Interface for MEDLINE/PubMed searches' (SLIM) which provided JavaScript slider bars to control search parameters. In this new version, recent developments in Web-based technologies were implemented. These changes may prove to be even more valuable in enhancing user interactivity through client-side manipulation and management of results.

**Results:**

PubMed Interact is a Web-based MEDLINE/PubMed search application built with HTML, JavaScript and PHP. It is implemented on a Windows Server 2003 with Apache 2.0.52, PHP 4.4.1 and MySQL 4.1.18. PHP scripts provide the backend engine that connects with E-Utilities and parses XML files. JavaScript manages client-side functionalities and converts Web pages into interactive platforms using dynamic HTML (DHTML), Document Object Model (DOM) tree manipulation and Ajax methods. With PubMed Interact, users can limit searches with JavaScript slider bars, preview result counts, delete citations from the list, display and add related articles and create relevance lists. Many interactive features occur at client-side, which allow instant feedback without reloading or refreshing the page resulting in a more efficient user experience.

**Conclusion:**

PubMed Interact is a highly interactive Web-based search application for MEDLINE/PubMed that explores recent trends in Web technologies like DOM tree manipulation and Ajax. It may become a valuable technical development for online medical search applications.

## Background

This research continues to investigate innovations in user-computer interface for online storage and retrieval systems in medical research. The goal of the project is to advance the development of a Web-based medical search tool that can enhance user interaction with the MEDLINE/PubMed database and push to the forefront the different strategies and filters in Entrez PubMed that often remain hidden from novice users, such as age groups, clinical study filters and systematic reviews. The long-term objective is to study and implement clean and effective user interfaces for MEDLINE/PubMed that increases utilization and improves search outcomes without overwhelming novice users and limiting the workflow of advanced users. This manuscript reports the development, implementation and technical evaluation of the research application, PubMed Interact.

An earlier version of this project is the Slider Interface for MEDLINE/PubMed searches, or SLIM [1]. SLIM is a Web-based application that implements JavaScript slider bars to set search limits and filters. It uses dynamic HTML (DHTML), which is the method of using static markup language and JavaScript to create interactive pages. Users can choose from several preset search parameters. They can hide and display abstracts when viewing the results. An educational feedback tool for MeSH terminologies called the 'information box' is also available.

New approaches in the development of Web-based applications prompted the exploration to look beyond search forms and provide users the ability to further interact with results. Document Object Model (DOM) tree manipulation and Ajax (Asynchronous JavaScript + XML) [2] have gained popularity and recognition among Web application developers. The Document Object Model is 'a platform- and language-neutral interface that permits scripts to access and update the content, structure, and style' of an HTML document [3]. Ajax provides a script engine for Web sites to send and receive small packets of data from the server without interrupting user activity. The combination of both methods can be a robust platform for alternative Web-based search applications for medical research.

PubMed Interact makes extensive use of DHTML, DOM tree manipulation and Ajax scripting to enhance interactivity and productivity. Although it extracts several features from SLIM, many of its integral features allow interactions with the retrieved set of citations. We hope this project will contribute to ongoing efforts to improve online storage and retrieval systems for medical literature.

## Implementation

### Search interface

**Figure 1:**
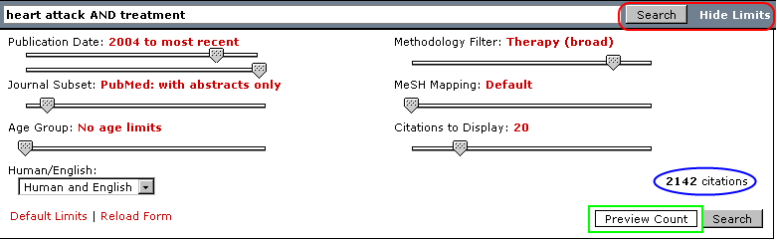
**PubMed Interact search form with search terms and slider settings**. Red rounded rectangle: Text link to hide slider limits beside the search button. Blue ellipse: Preview of the number of citations. Green box: Preview count button

PubMed Interact introduces an improved version of the SLIM interface [Figure 1]. A wide text box accepts input for search terms, while seven JavaScript slider bars and a dropdown menu control search limits and parameters. The search parameters include publication date, journal subset, age group, methodology filter, Medical Subject Headings (MeSH) mapping, human studies and language.

The publication date parameter uses two sliders: the start year slider and the end year slider. With an end year slider, searches are not limited to the current year as the permanent end date. Users can set different date ranges within the past 10 years, e.g. 1998 to 2002, or limit to one specific year, e.g. 2003. No publication date is set by default for the start year slider, i.e. no date limits. The end year slider defaults to the current year.

The PubMed database contains several subsets, among them the MEDLINE subset and the Core Clinical Journals. The journal subset slider controls options to search within the whole PubMed database, the MEDLINE database subset, or the Core Clinical Journals. Within each subset, users can further limit the search to articles with abstracts or to those with links to full-text or free full-text. The default setting searches the PubMed database without any abstract or full-text restrictions.

The age group slider is a modified version of the age group dropdown menu in the Limits page of Entrez PubMed. It reorders the limits according to age. It starts with 'Newborn' and ends with 'Adult Group'. No limits are set by default.

The methodology filter slider can limit searches to case reports, clinical study categories, or systematic reviews. The case report filter uses the publication type search tag of Entrez PubMed to limit the search. The clinical study categories, also called PubMed Clinical Queries, are 10 search methodology filters based on the works of Haynes RB et al [4]. The systematic reviews subset is a pre-configured filter that finds citations for systematic reviews, meta-analyses, reviews of clinical trials, evidence-based medicine, consensus development conferences, and guidelines [5]. No limits are set by default.

The MeSH mapping slider, a feature first developed in SLIM as the search mapping slider, is intended for intermediate to advance users of PubMed familiar with search tags and MeSH term operations. A customized PHP function extracts the mapped MeSH terms from the original search and modifies the search tags according to the slider setting. These modified terms are then appended to the current search to refine and redirect the search strategy. The default setting submits the search terms to the ESearch utility as entered in the text box without any modifications.

The last slider controls the number of citations to be displayed in the results list. It does not affect the search query. It merely provides users the option to display the number of retrieved citations (10, 20, 40, 60, 80 or 100).

To limit the search by subject or language, a dropdown menu below the sliders contains options for human studies, English language or both. To reload the form or reset the sliders to the default settings, users can click on the links found below the dropdown menu.

**Figure 2:**
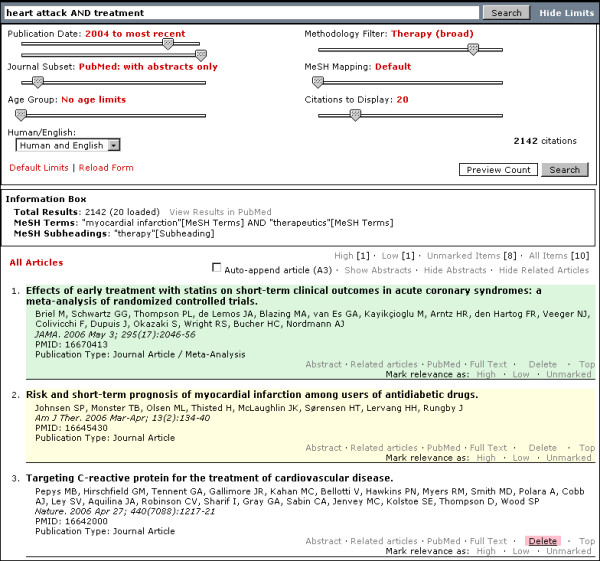
**PubMed Interact results interface with search limits displayed. Information box and list of citations are shown**. Light green background: Citation with user-tagged high relevance. Light yellow background: Citation with user-tagged low relevance. Light red background: Delete citation link.

**Figure 3:**
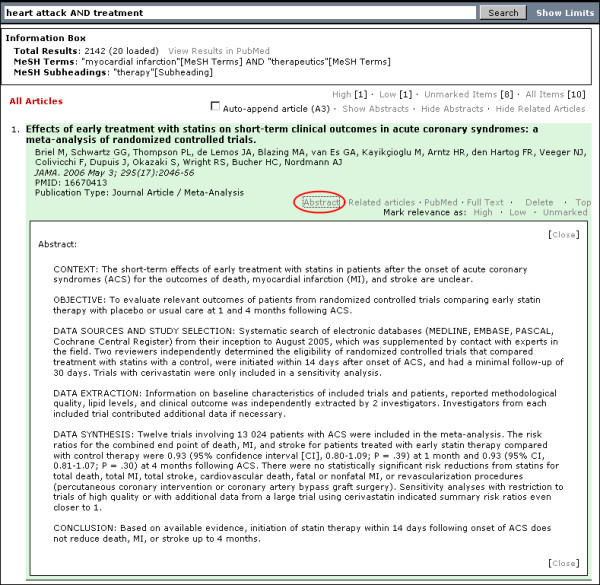
**PubMed Interact results interface with search limits hidden from view. Abstract of citation is displayed in formatted structure**. Red Ellipse: Text link to toggle display of abstract

Users can opt to hide the slider bars. A text link at the top right part of the search form beside the search button allows users to hide the search limits. This process is most advantageous when viewing the search results because it maximizes the display area of the browser page [Figure 2 and 3]. Scripting made use of dynamic HTML and DOM tree manipulation.

An interactive feature of the search form is the ability to preview the results count without submitting the form or reloading the page [Figure 1]. This function uses Ajax to fetch data from the server and DOM tree manipulation to display the resulting number of citations. After typing in the search terms and setting the limits, users can click on the 'Preview Count' button and the number of citations is displayed. This process can be repeated with different search terms and slider settings. This feature allows the user to quickly gauge the effectiveness of the keywords and search parameters before submitting the form.

### Results interface

PubMed Interact adapts two features from the results interface of SLIM: the information box and the ability to toggle the display of abstracts. An important distinction of PubMed Interact is the facility by which users can manipulate the search results.

The information box is displayed only after the search form is submitted [Figure 2 and 3]. Positioned below the search form, it provides the total number of results along with additional search information such as mapped MeSH Terms, mapped Subheadings and unmapped terms. It is intended as an educational tool for new users unfamiliar with the MeSH Thesaurus and as a search reference tool for advanced researchers.

Abstracts can be displayed or hidden from view [Figure 2 and 3]. It is possible to hide or display all abstracts as a group using links above the main results list. A link below the citation details will toggle the display for individual abstracts. An added feature in PubMed Interact is the ability to display structured abstracts. A simple PHP output function uses regular expressions to detect and display abstracts of a specific structure [Figure 3]. This facilitates reading and scanning of specific abstracts.

Removal of single citations from the search results is seldom found in Web-based medical search applications. In PubMed Interact, users can delete individual citations from the main list by clicking on a link below the citation details [Figure 2]. When a citation is deleted, it is highlighted with a light red background for a few seconds before it disappears from view and removed from the main HTML source code. The visual effect is achieved by DHTML, while removal of the citation from the HTML document is done through DOM tree manipulation. This delete function enables the user to keep only citations relevant to their search.

The 'Auto-Append Article' feature, also called A3, is linked with citation deletion. If active, the A3 function automatically retrieves the next citation in the results and appends it at the bottom of the list when a citation is deleted. The new citation data is retrieved from the local PMI domain server using Ajax scripting methods, while the action of appending and displaying that citation is done using DOM tree manipulation. All A3 processes are asynchronous and achieved without reloading the page. The appended article acquires the functionality of the original citations on the list. This feature is deactivated by default and can be activated using a checkbox at the top of the list.

PubMed Interact implements two relevance lists: high and low. These relevance lists are user-dependent and color-coded. Users can label specific citations according to relevance to the original search. Citations tagged with high relevance will have a light green background, while those with low relevance will have a light yellow background [Figure 2]. Citations without any labels will have the default white background. The relevance lists can be viewed separately using links found at the top of the main results list.

**Figure 4:**
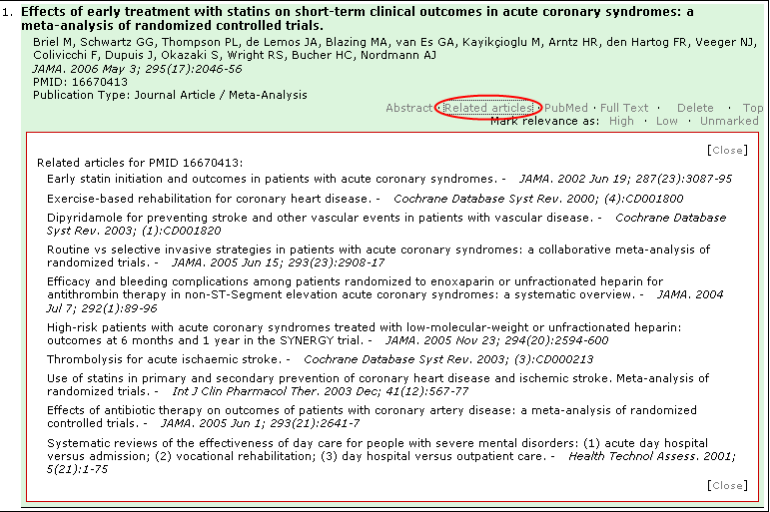
**Citation view showing top ten related articles**. Red Ellipse: Text link to load and toggle display of related articles.

**Figure 5:**
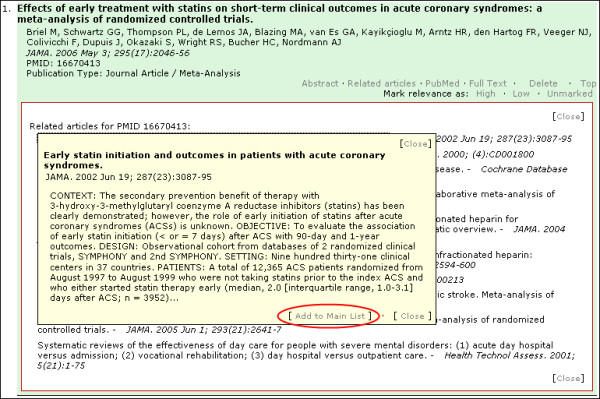
**Citation view with a floating HTML division element showing details of the related article**. Red Ellipse: Text link to add related article to the main results list.

An advanced interactive feature of PubMed Interact is the ability to retrieve the related articles of each citation within the same page. In the current PubMed Interact implementation, only the top 10 related articles are retrieved and displayed where the abstract is positioned [Figure 4]. Clicking on the title of a related article generates a floating HTML division element (DIV) with the citation details and an excerpt of the abstract [Figure 5]. The related article can be added to the main results list by clicking on the 'Add to Main List' link. This feature allows the user to change the composition of the main results list "on-the-fly" using related articles. The new citation is inserted immediately below the 'parent article' and acquires all the functionalities of the original citations on the list. This feature depends heavily on Ajax scripting methods, DOM tree manipulation, DHTML, JavaScript loops and server-side PHP functions.

### System development and implementation

PubMed Interact was developed on a server running Windows Server 2003 with Apache 2.0.52, PHP 4.4.1 and MySQL 4.1.18. The server-side scripts were coded in PHP and provided the backend engine of PubMed Interact. Client-side display and functionalities were written in HTML and JavaScript. The advanced and interactive features of PubMed Interact rely on DHTML, DOM tree manipulation and Ajax scripting methods, which were all written in JavaScript.

A large part of script development adopted the object-oriented programming (OOP) approach. A custom set of PHP classes connect to the Entrez Programming Utilities, specifically the ESearch, EFetch and ELink tools [6]. These PHP classes are modified XML parsers that send queries to E-Utilities and parse the retrieved XML files. The OOP approach allows developers to reuse sets of code for different functions, thus, drastically reducing the amount of code maintained and opening possibilities for expanding code functions. In PubMed Interact, the code used to get the citation details for the search is the same code used to get the details for the related articles.

The retrieved XML files are processed and stored in a local MySQL database to minimize the load on the E-Utilities servers. Instead of several remote queries to E-Utilities, the PHP scripts that retrieve data for the search results send one query and store the top 200 of the citation details regardless of the number of citations to be displayed. Thus, the A3 feature which appends new articles after a citation is deleted retrieves data from the local domain server and not from E-Utilities. The same process is used for the related articles of one citation. The details of all 10 related articles are stored in the local server and retrieved without reconnecting to the E-Utilities server.

JavaScript functions were essential in the development of client-side interactivity. DOM tree manipulation captured information within the page and passed the data to the Ajax script engine. The core of Ajax scripting is the XMLHTTPRequest Object of JavaScript which performs HTTP client functions. However, the XMLHTTPRequest Object, by design, can only connect to the local domain server and not to remote servers, e.g. E-Utilities. Thus, custom PHP scripts were written to receive data from the Ajax engine, connect to the E-Utilities server or to the local MySQL database as needed and deliver an output in HTML format. JavaScript then displays the output in the browser page using both the Ajax engine and DOM tree manipulation. This is all done asynchronously without reloading the page and interrupting user activity.

## Result and discussion

PubMed Interact is an experiment in user-computer interface. It is part of an ongoing project to make use of modern Web technologies in the development and improvement of Web-based medical search applications. The growing trend of using the Web as a platform to deliver services opens opportunities for alternative solutions in medical literature research. Web-based applications that function like traditional software, combined with rich user interface and improved user control of data, contribute to the indispensable nature of online information storage and retrieval systems for health resources.

Two important components of the trend are DOM tree manipulation and Ajax. By integrating both technologies, PubMed Interact bridges an effective search strategy with a highly-interactive interface. Users not only have the ability to modify searches by setting parameters, they can also label, delete and add from within the existing list of citations. Access to related articles in the same page also provides an additional resource for more relevant citations not found in the original search results.

The search interface of PubMed Interact exposes and facilitates the use of several search strategies available in Entrez PubMed. Some options in the Limits page of Entrez PubMed are available in the first four sliders, eliminating the need to go back and forth between pages to set search parameters. Two of the advanced search features of Entrez PubMed – the clinical study categories, also known as PubMed Clinical Queries, and the systematic reviews subset – are made available for both novice and seasoned users with the Methodology Filter slider. In Entrez PubMed, the MeSH terms and subheadings of a search are viewed from the Details tab. In PubMed Interact, the MeSH details mapped from the keywords are presented in the information box, which can then be used as guides for the MeSH Mapping slider. Several features for future integration may include adding publication types, language options and subsets and searching in the Journals and MeSH database. These efforts are consistent with the long-term aim of developing a user-computer interface for medical research that empowers novice users with interactive tools for search parameters and provides expert users with easy access to advanced search filters.

### Technical evaluation

The application is available online without restrictions. The alpha version and the beta version went live in late November 2005 and February 2006, respectively. The local MySQL database of the beta version contains over 29,900 records of citations in XML format and uses 54 megabytes of disk space. A scheduled maintenance script can be implemented in the future to delete old XML records from the database and keep the storage allocation manageable. This plan is deferred until the implementation is moved out of beta phase to record benchmarks for MySQL usage.

Browser compatibility evaluation showed full functionality in Windows versions of Mozilla Firefox 1.5+, Internet Explorer 5.5+ and Opera 8.5+ and in the Linux version of Mozilla Firefox 1.5+. Some formatting inconsistencies were observed in Mac OSX versions of Mozilla Firefox 1.5+ and Safari 2.0+ but no functionality problems were noted.

The search form and citation list of the application were tested using the W3C Markup Validation Service [7]. An unsupported element attribute for the Document Type Declaration used was reported for each slider. The validation report of the citation list accounted one recurring error for each citation. The error involves using numerical strings as id attributes for the citation divisions. Despite being reported as markup validation errors, these 'invalid codes' proved important for user-friendly functionality. They were also supported by the different browsers used for testing. Thus, they were noted down for reference but retained for use. Removing these 'invalid codes' degraded the functionality of the application.

### Limitations

This paper is limited to the development, implementation and technical evaluation of PubMed Interact. It does not provide empirical evidence to show increased efficiency in searching or better precision and recall for results. A formal user evaluation of the application is needed to validate the usability and benefits of an alternative PubMed search interface.

The technical evaluation of PubMed Interact employed commonly accepted procedures in Web applications, such as functionality, storage space used, markup validations and browser compatibility testing. It was not evaluated against any formal framework or standard criteria for software development.

### Future activities

User evaluation is valuable in the continued development of PubMed Interact. The researchers plan to do comparative studies between PubMed Interact and Entrez PubMed. Users with various levels of searching skills will perform structured and unstructured tasks. Through user interviews, online questionnaires and direct observation, the research team will assess the effectiveness of PubMed Interact as compared to Entrez PubMed in usability, performance and search outcomes. The educational impact, speed and stability of the system and the effect on searching attitudes and strategies will also be studied.

The projected study will be an opportunity to gather more information on how medical researchers interact with alternative search interfaces and obtain data on usability and functionality. User feedback will determine which features need to be improved or abandoned, and whether new functionalities should be added. As the progress of Web technologies continues, better platforms and methods will be available for further innovations in search interfaces for medical literature search.

## Conclusion

PubMed Interact is a Web-based MEDLINE/PubMed search application that explores recent trends in Web development technologies like DOM tree manipulation and Ajax scripting methods. Users can control search parameters, refocus search strategies and modify search results easily. Many enhanced and interactive features occur at client-side and allow instant feedback without reloading or refreshing the page. PubMed Interact is a novel approach in the development of online tools for medical information research.

## Availability and requirements

**Project name**: PubMed Interact

**Project home page**: 

**Operating systems**: Web-based, platform-independent

**Programming language**: PHP, JavaScript

**Other requirements**: JavaScript-enabled browsers, e.g. Fire Fox 1.0 or higher, IE 5.5 or higher

**License**: Free, anyone may use the service

**Any restrictions to use by non-academics**: None

## Abbreviations

**SLIM**: Slider Interface for MEDLINE/PubMed searches

**DHTML**: Dynamic HTML

**DOM**: Document Object Model

**Ajax**: Asynchronous JavaScript + XML

**XML**: Extensible Markup Language

**HTML**: HyperText Markup Language

**MeSH**: Medical Subject Headings

**PHP**: PHP: Hypertext Preprocessor

**A3**: Auto-Append Article

**OOP**: Object-oriented Programming

## Competing interests

The author(s) declare that they have no competing interests.

## Authors' contributions

MM conceived of the project, designed and developed the application and drafted the manuscript. PF assisted in the design and development of the application and reviewed the initial drafts of the manuscript. All authors read and approved the final manuscript.

## Pre-publication history

The pre-publication history for this paper can be accessed here:



## References

[B1] Muin M, Fontelo P, Liu F, Ackerman M SLIM: an alternative Web interface for MEDLINE/PubMed searches – a preliminary study. BMC Med Inform Decis Mak.

[B2] Adaptive Path – Ajax: A New Approach to Web Applications. http://www.adaptivepath.com/publications/essays/archives/000385.php.

[B3] W3C Document Object Model. http://www.w3.org/DOM/.

[B4] Summary of Enhancements for Clinical Queries for MEDLINE for Studies. http://www.nlm.nih.gov/pubs/techbull/jf04/cq_info.html.

[B5] Search Strategy Used to Create the Systematic Reviews Subset on PubMed. http://www.nlm.nih.gov/bsd/pubmed_subsets/sysreviews_strategy.html.

[B6] Entrez Programming Utilities. http://eutils.ncbi.nlm.nih.gov/entrez/query/static/eutils_help.html.

[B7] W3C Markup Validation Service. http://validator.w3.org.

[B8] WebFX. http://webfx.eae.net.

[B9] Dynamic Drive. http://www.dynamicdrive.com.

